# Diabetes-related distress and its associated factors among patients with type 2 diabetes mellitus in Tunisia

**DOI:** 10.1192/j.eurpsy.2023.1631

**Published:** 2023-07-19

**Authors:** R. Masmoudi, M. Bouattour, F. Hadj Kacem, R. Ben Jemaa, F. Cherif, J. Masmoudi, M. Abid

**Affiliations:** 1 Psychiatry; 2 Endocrinology, Hedi Chaker University Hospital, Sfax, Tunisia

## Abstract

**Introduction:**

Diabetes-related distress (DD) is one of the psychological disorders affecting patients with diabetes.

**Objectives:**

The aim of this study was to assess the prevalence and level of DD and its associated factors among patients with type 2 diabetes.

**Methods:**

This was descriptive and analytical cross-sectional study, carried out with patients followed for type 2 diabetes at the endocrinology consultation.

The participant’s sociodemographic and clinical information was obtained through face-to-face interviews and medical records.

DD was assessed using the Arabic version of diabetes distress scale (DDS-17). The DDS contains 17 items, each rated on a 6-point Likert scale. The scale yields a total diabetes distress score, and scores for four subscales: emotional burden, regimen distress, physician distress and interpersonal distress.

**Results:**

There were 103 subjects. The mean age was 59.31 ±10.83 years with a sex ratio (M/F) = 1.19.

Median duration of diabetes was 7 years **(IQR 3 ; 12** years). Among our patients, 31.1% of patients had properly controlled diabetes (HbA1c < 7%) and 41% had at least one diabetes complication.

The prevalence of diabetes related distress was 70.90% in which emotional distress was the most prevalent (78.60%) domain.

Low socio-economic level (p=0.001), married status (p=0.034) having diabetes complications (p=0.008) younger age at onset of diabetes (p=0.001) were associated with diabetes related distress. Poor HbA1c control (HbA1c≥7%) was significantly correlated with DD (p≤0,001).

**Conclusions:**

Our study suggests that diabetes related distress was highly prevalent in type 2 diabetes patients in Tunisia. Active screening for DD should be an integral part of diabetes care.

**Disclosure of Interest:**

None Declared

